# Supplementary material for: Genetic analysis of the Arabidopsis TIR1/AFB auxin receptors reveals both overlapping and specialized functions
Source: eLife. 2020 Feb 18;9:e54740. doi: 10.7554/eLife.54740 (PMC7048394; doi:10.7554/eLife.54740)
Supplement: Supplementary file 3. [file elife-54740-supp3.docx]

*Aethionema arabicum* https://genomevolution.org/coge/GenomeInfo.pl?gid=36061 (Haudry et al. 2013; Nguyen et al. 2019)

*Arabidopsis thaliana* (AraPort11) <https://www.araport.org> (Cheng et al. 2017)

*Arabis alpina* (v5) <https://www.ncbi.nlm.nih.gov/genome/17813> (Willing et al. 2015)

*Azolla filiculoides* (v1.1) <https://www.fernbase.org> (Li et al. 2018)

*Camelina sativa* (v2) <http://www.camelinadb.ca/downloads.html> (Kagale et al. 2014)

*Cardamine hirsuta* (v1) <http://chi.mpipz.mpg.de/index.html> (Gan et al. 2016)

*Klebsormidium nitens* (formerly K. flaccidum) (v1.0) <http://www.plantmorphogenesis.bio.titech.ac.jp/~algae_genome_project/klebsormidium/index.html> (Hori et al. 2014)

*Leavenworthia alabamica* <https://www.ncbi.nlm.nih.gov/assembly/GCA_000411055.1/> (Haudry et al. 2013)

*Phoenix dactylifera* (PDK20) <http://qatar-weill.cornell.edu/research/research-highlights/date-palm-research-program/date-palm-draft-sequence> (Al-Mssallem et al. 2013)

*Pinus taeda* (pita2.01) <https://treegenesdb.org/FTP/Genomes/Pita/> (Zimin et al. 2014)

*Pohlia nutans* (GenBank TSA) (Liu et al. 2013)

*Salvinia cucullata* (v1.2) <https://www.fernbase.org> (Li et al. 2018)

*Tarenaya hassleriana* (v1) <https://www.ncbi.nlm.nih.gov/assembly/GCF_000463585.1/> (Cheng et al. 2013)

*Thlaspi arvense* (v1.0) <http://pennycress.umn.edu> (Dorn et al. 2015)

https://phytozome.jgi.doe.gov/pz/portal.html (Goodstein et al. 2012)

*Amborella trichopoda* (v1.0) (Amborella Genome 2013)

*Ananas comosus* (v3) (Ming et al. 2015)

*Aquilegia coerulea* (v3.1) (Filiault et al. 2018)

*Arabidopsis lyrata* (v2.1) (Hu et al. 2011)

*Arabidopsis halleri* (v1.1)

*Boechera stricta* (v1.2) (Lee et al. 2017)

*Brassica rapa* (v1.3)

*Capsella rubella* (v1) (Slotte et al. 2013)

*Carica papaya* (v0.4) (Ming et al. 2008)

*Ceratodon purpureus* (R40 transcriptome)

*Citrus sinensis* (v1.1) (Wu et al. 2014)

*Cucumis sativus* (v1.0)

*Daucus carota* (v2.0) (Iorizzo et al. 2016)

*Eucalyptus grandis* (v2.0) (Myburg et al. 2014)

*Eutrema salsugineum* (v1.0) (Yang et al. 2013)

*Fragaria vesca* (v1.1) (Shulaev et al. 2011)

*Gossypium raimondii* (v2.1) (Paterson et al. 2012)

*Marchantia polymorpha* (v3.1) (Bowman et al. 2017)

*Medicago truncatula* (4.0v1) (Young et al. 2011)

*Mimulus guttatus* (v2.0) (Hellsten et al. 2013)

*Oryza sativa* (v7.0) (Ouyang et al. 2007)

*Physcomitrella patens* (v3.3) (Lang et al. 2018)

*Populus trichocarpa* (v3.1) (Tuskan et al. 2006)

*Prunus persica* (v2.1) (Verde et al. 2017)

*Ricinus communis* (v0.1) (Chan et al. 2010)

*Selaginella moellendorffii* (v1.0) (Banks et al. 2011)

*Solanum lycopersicum* (iTAG2.4) (Tomato Genome 2012)

*Sphagnum fallax* (v0.5)

*Theobroma cacao* (v1.1) (Motamayor et al. 2013)

*Vitis vinifera* (Genoscope.12X) (Jaillon et al. 2007)

*Zea mays* (Ensembl-18) (Schnable et al. 2009)

http://www.onekp.com/public_data.html (<https://db.cngb.org/blast4onekp>) (Johnson et al. 2012; Matasci et al. 2014; Wickett et al. 2014; Xie et al. 2014; One Thousand Plant Transcriptomes 2019)

*Adiantum tenerum* (BMJR)

*Angiopteris evecta* (NHCM)

*Aristolochia elegans* (PAWA)

*Cochlearia officinalis* (CSUV)

*Coleochaete irregularis* (QPDY)

*Coleochaete scutata* (VQBJ)

*Cycas micholitzii* (XZUY)

*Cylindrocystis brebissonii* (YOXI)

*Cylindrocystis cushleckae* (JOJQ)

*Cylindrocystis* sp (VAZE)

*Diphasiastrum digitatum* (WAFT)

*Equisetum hymale* (JVSZ)

*Gyrostemon ramulosus* (UAXP)

*Huperzia squarrosa* (GAON)

*Illicium floridanum* (VZCI)

*Kadsura heteroclite* (NWMY)

*Ludovia* sp (VVVV)

*Lygodium japonicum* (PBUU)

*Megaceros vincentianus* (TCBC)

*Moringa oleifera* (CZPV)

*Nothoceros aenigmaticus* (DXOU)

*Nymphaea* sp (PZRT)

*Persea borbonia* (WIGA)

*Podocarpus rubens* (XLGK)

*Polanisia trachysperma* (QSKP)

*Polypodium glycyrrhiza* (CJNT)

*Polypodium hesperium* (ZRAV)

*Polytrichum commune* (SZYG)

*Porella navicularis* (KRUQ)

*Psilotum nudum* (QVMR)

*Roya obtusa* (XRTZ)

*Salvadora* sp. (RTTY)

*Sceptridium dissectum* (EEAQ)

*Selaginella acanthonota* (ZYCD)

*Takakia lepidozioides* (SKQD)

*Tetrastigma voinierianum* (SZPD)

*Tropaeolum peregrinum* (MYZV)

*Woodsia scopulina* (YJJY)

Floral Genome Project (Jiao et al. 2011)

*Liriodendron tulipifera* (FGP-build3)

*Nuphar advena* (FGP-build3)

**SUPPLEMENTAL REFERENCES**

Al-Mssallem IS, Hu S, Zhang X, Lin Q, Liu W, Tan J, Yu X, Liu J, Pan L, Zhang T et al. 2013. Genome sequence of the date palm *Phoenix dactylifera* L. *Nat Commun* **4**: 2274.

Amborella Genome P. 2013. The *Amborella* genome and the evolution of flowering plants. *Science* **342**: 1241089.

Banks JA Nishiyama T Hasebe M Bowman JL Gribskov M dePamphilis C Albert VA Aono N Aoyama T Ambrose BA et al. 2011. The *Selaginella* genome identifies genetic changes associated with the evolution of vascular plants. *Science* **332**: 960-963.

Bowman JL Kohchi T Yamato KT Jenkins J Shu S Ishizaki K Yamaoka S Nishihama R Nakamura Y Berger F et al. 2017. Insights into Land Plant Evolution Garnered from the *Marchantia polymorpha* Genome. *Cell* **171**: 287-304 e215.

Chan AP, Crabtree J, Zhao Q, Lorenzi H, Orvis J, Puiu D, Melake-Berhan A, Jones KM, Redman J, Chen G et al. 2010. Draft genome sequence of the oilseed species *Ricinus communis*. *Nat Biotechnol* **28**: 951-956.

Cheng CY, Krishnakumar V, Chan AP, Thibaud-Nissen F, Schobel S, Town CD. 2017. Araport11: a complete reannotation of the *Arabidopsis thaliana* reference genome. *Plant J* **89**: 789-804.

Cheng S, van den Bergh E, Zeng P, Zhong X, Xu J, Liu X, Hofberger J, de Bruijn S, Bhide AS, Kuelahoglu C et al. 2013. The *Tarenaya hassleriana* genome provides insight into reproductive trait and genome evolution of crucifers. *Plant Cell* **25**: 2813-2830.

Dorn KM, Fankhauser JD, Wyse DL, Marks MD. 2015. A draft genome of field pennycress (*Thlaspi arvense*) provides tools for the domestication of a new winter biofuel crop. *DNA Res* **22**: 121-131.

Filiault DL, Ballerini ES, Mandakova T, Akoz G, Derieg NJ, Schmutz J, Jenkins J, Grimwood J, Shu S, Hayes RD et al. 2018. The *Aquilegia* genome provides insight into adaptive radiation and reveals an extraordinarily polymorphic chromosome with a unique history. *Elife* **7**.

Gan X, Hay A, Kwantes M, Haberer G, Hallab A, Ioio RD, Hofhuis H, Pieper B, Cartolano M, Neumann U et al. 2016. The *Cardamine hirsuta* genome offers insight into the evolution of morphological diversity. *Nat Plants* **2**: 16167.

Goodstein DM, Shu S, Howson R, Neupane R, Hayes RD, Fazo J, Mitros T, Dirks W, Hellsten U, Putnam N et al. 2012. Phytozome: a comparative platform for green plant genomics. *Nucleic Acids Res* **40**: D1178-1186.

Haudry A, Platts AE, Vello E, Hoen DR, Leclercq M, Williamson RJ, Forczek E, Joly-Lopez Z, Steffen JG, Hazzouri KM et al. 2013. An atlas of over 90,000 conserved noncoding sequences provides insight into crucifer regulatory regions. *Nat Genet* **45**: 891-898.

Hellsten U, Wright KM, Jenkins J, Shu S, Yuan Y, Wessler SR, Schmutz J, Willis JH, Rokhsar DS. 2013. Fine-scale variation in meiotic recombination in *Mimulus* inferred from population shotgun sequencing. *Proc Natl Acad Sci U S A* **110**: 19478-19482.

Hori K, Maruyama F, Fujisawa T, Togashi T, Yamamoto N, Seo M, Sato S, Yamada T, Mori H, Tajima N et al. 2014. *Klebsormidium flaccidum* genome reveals primary factors for plant terrestrial adaptation. *Nat Commun* **5**: 3978.

Hu TT, Pattyn P, Bakker EG, Cao J, Cheng JF, Clark RM, Fahlgren N, Fawcett JA, Grimwood J, Gundlach H et al. 2011. The *Arabidopsis lyrata* genome sequence and the basis of rapid genome size change. *Nat Genet* **43**: 476-481.

Iorizzo M, Ellison S, Senalik D, Zeng P, Satapoomin P, Huang J, Bowman M, Iovene M, Sanseverino W, Cavagnaro P et al. 2016. A high-quality carrot genome assembly provides new insights into carotenoid accumulation and asterid genome evolution. *Nat Genet* **48**: 657-666.

Jaillon O, Aury JM, Noel B, Policriti A, Clepet C, Casagrande A, Choisne N, Aubourg S, Vitulo N, Jubin C et al. 2007. The grapevine genome sequence suggests ancestral hexaploidization in major angiosperm phyla. *Nature* **449**: 463-467.

Jiao Y, Wickett NJ, Ayyampalayam S, Chanderbali AS, Landherr L, Ralph PE, Tomsho LP, Hu Y, Liang H, Soltis PS et al. 2011. Ancestral polyploidy in seed plants and angiosperms. *Nature* **473**: 97-100.

Johnson MT, Carpenter EJ, Tian Z, Bruskiewich R, Burris JN, Carrigan CT, Chase MW, Clarke ND, Covshoff S, Depamphilis CW et al. 2012. Evaluating methods for isolating total RNA and predicting the success of sequencing phylogenetically diverse plant transcriptomes. *PLoS One* **7**: e50226.

Kagale S, Koh C, Nixon J, Bollina V, Clarke WE, Tuteja R, Spillane C, Robinson SJ, Links MG, Clarke C et al. 2014. The emerging biofuel crop *Camelina sativa* retains a highly undifferentiated hexaploid genome structure. *Nat Commun* **5**: 3706.

Lang D, Ullrich KK, Murat F, Fuchs J, Jenkins J, Haas FB, Piednoel M, Gundlach H, Van Bel M, Meyberg R et al. 2018. The *Physcomitrella patens* chromosome-scale assembly reveals moss genome structure and evolution. *Plant J* **93**: 515-533.

Lee CR, Wang B, Mojica JP, Mandakova T, Prasad K, Goicoechea JL, Perera N, Hellsten U, Hundley HN, Johnson J et al. 2017. Young inversion with multiple linked QTLs under selection in a hybrid zone. *Nat Ecol Evol* **1**: 119.

Li FW, Brouwer P, Carretero-Paulet L, Cheng S, de Vries J, Delaux PM, Eily A, Koppers N, Kuo LY, Li Z et al. 2018. Fern genomes elucidate land plant evolution and cyanobacterial symbioses. *Nat Plants* **4**: 460-472.

Liu S, Wang N, Zhang P, Cong B, Lin X, Wang S, Xia G, Huang X. 2013. Next-generation sequencing-based transcriptome profiling analysis of *Pohlia nutans* reveals insight into the stress-relevant genes in Antarctic moss. *Extremophiles* **17**: 391-403.

Matasci N, Hung LH, Yan Z, Carpenter EJ, Wickett NJ, Mirarab S, Nguyen N, Warnow T, Ayyampalayam S, Barker M et al. 2014. Data access for the 1,000 Plants (1KP) project. *Gigascience* **3**: 17.

Ming R, Hou S, Feng Y, Yu Q, Dionne-Laporte A, Saw JH, Senin P, Wang W, Ly BV, Lewis KL et al. 2008. The draft genome of the transgenic tropical fruit tree papaya (*Carica papaya* Linnaeus). *Nature* **452**: 991-996.

Ming R, VanBuren R, Wai CM, Tang H, Schatz MC, Bowers JE, Lyons E, Wang ML, Chen J, Biggers E et al. 2015. The pineapple genome and the evolution of CAM photosynthesis. *Nat Genet* **47**: 1435-1442.

Motamayor JC, Mockaitis K, Schmutz J, Haiminen N, Livingstone D, 3rd, Cornejo O, Findley SD, Zheng P, Utro F, Royaert S et al. 2013. The genome sequence of the most widely cultivated cacao type and its use to identify candidate genes regulating pod color. *Genome Biol* **14**: r53.

Myburg AA, Grattapaglia D, Tuskan GA, Hellsten U, Hayes RD, Grimwood J, Jenkins J, Lindquist E, Tice H, Bauer D et al. 2014. The genome of *Eucalyptus grandis*. *Nature* **510**: 356-362.

Nguyen TP, Muhlich C, Mohammadin S, van den Bergh E, Platts AE, Haas FB, Rensing SA, Schranz ME. 2019. Genome Improvement and Genetic Map Construction for *Aethionema arabicum*, the First Divergent Branch in the Brassicaceae Family. *G3 (Bethesda)* **9**: 3521-3530.

One Thousand Plant Transcriptomes I. 2019. One thousand plant transcriptomes and the phylogenomics of green plants. *Nature* **574**: 679-685.

Ouyang S, Zhu W, Hamilton J, Lin H, Campbell M, Childs K, Thibaud-Nissen F, Malek RL, Lee Y, Zheng L et al. 2007. The TIGR Rice Genome Annotation Resource: improvements and new features. *Nucleic Acids Res* **35**: D883-887.

Paterson AH, Wendel JF, Gundlach H, Guo H, Jenkins J, Jin D, Llewellyn D, Showmaker KC, Shu S, Udall J et al. 2012. Repeated polyploidization of *Gossypium* genomes and the evolution of spinnable cotton fibres. *Nature* **492**: 423-427.

Schnable PS Ware D Fulton RS Stein JC Wei F Pasternak S Liang C Zhang J Fulton L Graves TA et al. 2009. The B73 maize genome: complexity, diversity, and dynamics. *Science* **326**: 1112-1115.

Shulaev V, Sargent DJ, Crowhurst RN, Mockler TC, Folkerts O, Delcher AL, Jaiswal P, Mockaitis K, Liston A, Mane SP et al. 2011. The genome of woodland strawberry (*Fragaria vesca*). *Nat Genet* **43**: 109-116.

Slotte T, Hazzouri KM, Agren JA, Koenig D, Maumus F, Guo YL, Steige K, Platts AE, Escobar JS, Newman LK et al. 2013. The *Capsella rubella* genome and the genomic consequences of rapid mating system evolution. *Nat Genet* **45**: 831-835.

Tomato Genome C. 2012. The tomato genome sequence provides insights into fleshy fruit evolution. *Nature* **485**: 635-641.

Tuskan GA Difazio S Jansson S Bohlmann J Grigoriev I Hellsten U Putnam N Ralph S Rombauts S Salamov A et al. 2006. The genome of black cottonwood, *Populus trichocarpa* (Torr. & Gray). *Science* **313**: 1596-1604.

Verde I, Jenkins J, Dondini L, Micali S, Pagliarani G, Vendramin E, Paris R, Aramini V, Gazza L, Rossini L et al. 2017. The Peach v2.0 release: high-resolution linkage mapping and deep resequencing improve chromosome-scale assembly and contiguity. *BMC Genomics* **18**: 225.

Wickett NJ, Mirarab S, Nguyen N, Warnow T, Carpenter E, Matasci N, Ayyampalayam S, Barker MS, Burleigh JG, Gitzendanner MA et al. 2014. Phylotranscriptomic analysis of the origin and early diversification of land plants. *Proc Natl Acad Sci U S A* **111**: E4859-4868.

Willing EM, Rawat V, Mandakova T, Maumus F, James GV, Nordstrom KJ, Becker C, Warthmann N, Chica C, Szarzynska B et al. 2015. Genome expansion of *Arabis alpina* linked with retrotransposition and reduced symmetric DNA methylation. *Nat Plants* **1**: 14023.

Wu GA, Prochnik S, Jenkins J, Salse J, Hellsten U, Murat F, Perrier X, Ruiz M, Scalabrin S, Terol J et al. 2014. Sequencing of diverse mandarin, pummelo and orange genomes reveals complex history of admixture during citrus domestication. *Nat Biotechnol* **32**: 656-662.

Xie Y, Wu G, Tang J, Luo R, Patterson J, Liu S, Huang W, He G, Gu S, Li S et al. 2014. SOAPdenovo-Trans: de novo transcriptome assembly with short RNA-Seq reads. *Bioinformatics* **30**: 1660-1666.

Yang R, Jarvis DE, Chen H, Beilstein MA, Grimwood J, Jenkins J, Shu S, Prochnik S, Xin M, Ma C et al. 2013. The Reference Genome of the Halophytic Plant *Eutrema salsugineum*. *Front Plant Sci* **4**: 46.

Young ND Debelle F Oldroyd GE Geurts R Cannon SB Udvardi MK Benedito VA Mayer KF Gouzy J Schoof H et al. 2011. The *Medicago* genome provides insight into the evolution of rhizobial symbioses. *Nature* **480**: 520-524.

Zimin A, Stevens KA, Crepeau MW, Holtz-Morris A, Koriabine M, Marcais G, Puiu D, Roberts M, Wegrzyn JL, de Jong PJ et al. 2014. Sequencing and assembly of the 22-gb loblolly pine genome. *Genetics* **196**: 875-890.
