# Supplementary material for: Genetic analysis of the Arabidopsis TIR1/AFB auxin receptors reveals both overlapping and specialized functions
Source: eLife. 2020 Feb 18;9:e54740. doi: 10.7554/eLife.54740 (PMC7048394; doi:10.7554/eLife.54740)
Supplement: Transparent reporting form [file elife-54740-transrepform.second.docx]

***eLife’s* transparent reporting form**

We encourage authors to provide detailed information *within their submission* to facilitate the interpretation and replication of experiments. Authors can upload supporting documentation to indicate the use of appropriate reporting guidelines for health-related research (see [EQUATOR Network](http://www.equator-network.org/%20)), life science research (see the [BioSharing Information Resource](https://biosharing.org/)), or the [ARRIVE guidelines](http://www.plosbiology.org/article/info:doi/10.1371/journal.pbio.1000412) for reporting work involving animal research. Where applicable, authors should refer to any relevant reporting standards documents in this form.

If you have any questions, please consult our Journal Policies and/or contact us: [editorial@elifesciences.org](mailto:editorial@elifesciences.org).

**Sample-size estimation**

- You should state whether an appropriate sample size was computed when the study was being designed
- You should state the statistical method of sample size computation and any required assumptions
- If no explicit power analysis was used, you should describe how you decided what sample (replicate) size (number) to use

Please outline where this information can be found within the submission (e.g., sections or figure legends), or explain why this information doesn’t apply to your submission:

Sample sizes are indicated in the legends for Figure 3, Figure 7, Figure 1—supplements 2, 4, 5, Figure 5—supplement 1, Figure 6—supplement 4, and Figure 7—supplement 1. Sample sizes for Figure 5, Figure 6, and Figure 6—figure supplement 1 are in the panels. The sample sizes in Figure 3—supplement 3 are included in the table.

**Replicates**

- You should report how often each experiment was performed
- You should include a definition of biological versus technical replication
- The data obtained should be provided and sufficient information should be provided to indicate the number of independent biological and/or technical replicates
- If you encountered any outliers, you should describe how these were handled
- Criteria for exclusion/inclusion of data should be clearly stated
- High-throughput sequence data should be uploaded before submission, with a private link for reviewers provided (these are available from both GEO and ArrayExpress)

Please outline where this information can be found within the submission (e.g., sections or figure legends), or explain why this information doesn’t apply to your submission:

Seedlings that became detached from the medium or whose roots grew away from media on the vertical plates were excluded from root elongation assays. For Figure 1—supplement 4, two soil-grown plants were excluded because their meristems arrested after transplanting and one was excluded after genotyping. In Figure 6—figure supplement 4, one afb1345 hypocotyl crossed over another so both were excluded.

**Statistical reporting**

- Statistical analysis methods should be described and justified
- Raw data should be presented in figures whenever informative to do so (typically when N per group is less than 10)
- For each experiment, you should identify the statistical tests used, exact values of N, definitions of center, methods of multiple test correction, and dispersion and precision measures (e.g., mean, median, SD, SEM, confidence intervals; and, for the major substantive results, a measure of effect size (e.g., Pearson's r, Cohen's d)
- Report exact p-values wherever possible alongside the summary statistics and 95% confidence intervals. These should be reported for all key questions and not only when the p-value is less than 0.05.

Please outline where this information can be found within the submission (e.g., sections or figure legends), or explain why this information doesn’t apply to your submission:

The statistical tests are described in the figure legends and/or in the text. Two-tailed *t*-tests were used for data in Figure 3N–O, Figure 5S, Figure 7, Figure 1—figure supplement 1B, Figure 5—figure supplement 1, Figure 6—figure supplement 4, and Figure 7—figure supplement 1. Fisher’s exact tests (2×2 table) were used for data in Figure 3P and Figure 1—figure supplement 5. A one-way ANOVA coupled with post hoc Tukey honestly significant difference (HSD) test was used for data in Figure 6. A Chi-squared test was used in Figure 3—figure supplement 3. Supplementary File 5 contains all pairwise statistical tests.

(For large datasets, or papers with a very large number of statistical tests, you may upload a single table file with tests, Ns, etc., with reference to sections in the manuscript.)

**Group allocation**

- Indicate how samples were allocated into experimental groups (in the case of clinical studies, please specify allocation to treatment method); if randomization was used, please also state if restricted randomization was applied
- Indicate if masking was used during group allocation, data collection and/or data analysis

Please outline where this information can be found within the submission (e.g., sections or figure legends), or explain why this information doesn’t apply to your submission:

For root elongation assays, the seedlings for different genotypes were grown in different quadrants of 4 plates for most experiments or in the top row/bottom rows of two plates for Figure 1—figure supplement 4E and Figure 5—figure supplement 1. Plates were shifted to different positions in the chamber shelf daily. The five plants per genotype for Figure 1—figure supplement 4A-C were all in different positions in the chamber.

**Additional data files (“source data”)**

- We encourage you to upload relevant additional data files, such as numerical data that are represented as a graph in a figure, or as a summary table
- Where provided, these should be in the most useful format, and they can be uploaded as “Source data” files linked to a main figure or table
- Include model definition files including the full list of parameters used
- Include code used for data analysis (e.g., R, MatLab)
- Avoid stating that data files are “available upon request”

Please indicate the figures or tables for which source data files have been provided:

An Excel file containing phenotypic measurements will be uploaded.
